# Rheumatology in the Middle East in 2017: clinical challenges and research

**DOI:** 10.1186/s13075-017-1359-0

**Published:** 2017-06-30

**Authors:** Abdulla Watad, Jamal Al-Saleh, Merav Lidar, Howard Amital, Yehuda Shoenfeld

**Affiliations:** 1 Department of Medicine ‘B’, Sheba Medical Center, Tel-Hashomer, Israel; 2 The Zabludowicz Center for Autoimmune Diseases, Sheba Medical Center, Tel-Hashomer, 5265601 Israel; 3 Rheumatology Unit, Sheba Medical Center, Tel-Hashomer, Israel; 4 Sackler Faculty of Medicine, Tel-Aviv University, Tel Aviv, Israel; 5 Rheumatology Unit, Dubai Hospital, Dubai, United Arab Emirates

**Keywords:** Rheumatology, FMF, Behçet’s disease, ASIA syndrome, Israel, Middle East

## Abstract

The World Health Organization (WHO) has ranked musculoskeletal diseases among the top ten leading causes of disability in the Middle East. The situation in the Middle East is unique as, although seventeen countries are geographical connected, there is considerable variability in the standard of rheumatology care and research between the countries. There are several factors contributing to this variability: allocated government resources to healthcare services, country demographics, the implemented healthcare system, the number of qualified rheumatologist, political stability, and the immigration to and from these countries.

## Clinical challenges in the Middle East

### Israel

In Israel, despite being one of the most developed countries in the Middle East, there are still many clinically challenging issues frequently encountered, including that the number of board-certified rheumatologists is approximately 150, thus making the waiting time until an appointment often too long. In some areas of the country, the time until the initial visit to a rheumatologist might exceed 3 months. In addition, regarding inflammatory disorders, there are still formal barriers to the rapid use of biologicals; for instance, in order to be entitled to biological use, rheumatoid arthritis patients need to fail on three oral disease-modifying antireheumatic drugs (DMARDS), which includes methotrexate. These local guidelines are economically driven and have not been updated since 2003.

### Other Middle East countries

#### Health systems

Government expenditure on healthcare services influences the practice of rheumatology in the Middle East. In the less prosperous countries, there is limited access to rheumatologists and treatment. On the other hand, in some booming countries the government predominately funds rheumatology services or has fully implemented a health insurance system that provides the same coverage. This grants easy access to rheumatologists and treatment. The stable environment and work opportunities in these countries in the Middle East have attracted millions of immigrants [1]. Hence, governments responded by reforming the healthcare system by introducing healthcare insurance to everyone, encouraging private sector growth, and taking the initial steps in monitoring the standard of care provided to the people living in those countries. In general, rheumatologists in the Middle East adopt and implement international standards of care in their daily practice [2]. The development of registries for different rheumatic diseases also helps in understanding real-life data and has improved patient care in the Middle East [3].

There is an opportunity for optimizing the access to rheumatology care in those countries. It is the role of the key opinion leaders in the Middle East to enlighten the decision makers of the growing burden of rheumatic diseases and the cost-effective outcomes of providing optimum rheumatology care to their nation in the long run.

#### Workforce

The postgraduate training in Western countries and the availability of training programs in different Middle East countries have helped in increasing the number of practicing rheumatologist in the last few decades. However, the current number is insufficient compared to developed countries. The ratio of rheumatologists per 100,000 population ranges from 0.3 to 0.89 [4]. The lack of pediatric rheumatologists is even worse. In addition, the massive immigration of qualified physicians, including rheumatologists, from the troubled counties has further worsened the rheumatology care in those countries.

Given the current burden of musculoskeletal diseases and the expected increase in the near future, health authorities in the Middle East should include a rheumatology specialty in their workforce planning. Substantial measures should be directed to undergraduate and postgraduate training to attract students and newly qualified physicians to the rheumatology specialty.

## Research in the Middle East

### Israel

Ben Zvi et al. [5] from the Sheba Medical Center have conducted the first randomized placebo-controlled trial of anakinra in colchicine-resistant familial Mediterranean fever (FMF) showing that the interleukin (IL)-1 receptor antagonist is useful in reducing attack frequency and improving the quality of life in patients with colchicine-resistant FMF.

These authors have also collaborated with colleagues from Turkey and Italy [6] in defining a severity score for FMF which will serve as a useful tool in assessing the utility of therapeutic interventions in clinical trials in both children and adults.

Moving on from FMF to other rheumatic diseases, Ofer-Shiber and Molad at the Rabin Medical Center in Petach-Tikva reported that a C-reactive peptide (CRP) level ≥0.9 mg/dl at diagnosis in patients with psoriatic arthritis predicted an earlier need for tumor necrosis factor (TNF) blockers in order to achieve disease control [7]. The role of vitamin D in systemic lupus erythematosus (SLE) has been investigated at the Sheba Medical Center showing, among other things, a higher incidence of hypocalcemic events among SLE patients in comparison with controls. Colafrancesco et al. have recently reported an association between ‘autoimmune/inflammatory syndrome induced by adjuvant’ (ASIA) and Sjogren’s syndrome on top of an overlap in some clinical manifestations such as dry mouth and dry eyes [8]. The group led by Amital and colleagues used “big data” analysis to investigate the association between comorbidities and how medical interventions affect outcomes over time [9, 10].

In Tel-Aviv, at the Sourasky Medical Center, Ablin et al. reported that magnetic resonance imaging (MRI) findings consistent with sacroiliitis were found among a significant proportion of patients diagnosed with primary fibromyalgia [11]. The same group described the use of induced sputum analysis in patients diagnosed with systemic sclerosis showing changes in cellular pattern and correlation with relevant clinical and pulmonary function parameters. At the Soroka Medical Center in Beer-Sheva‬, Abu-Shakra found no association between SLE disease activity and depression [12].‬‬‬‬‬

At the Rambam Medical Center in Haifa, Balbir and collaborators from the EUSTAR group observed that, in patients with systemic sclerosis that present early after the onset of Raynaud’s phenomenon, the manifestation of 50% of all incident organs occurs within 2 years simultaneously, rather than sequentially [13].

Zisman, from the Carmel Medical Center in Haifa, reported the risk of herpes zoster was significantly increased with age, treatment with steroids, and combination of antiTNF-α agents and c-DMARDs, but not with c-DMARDs or antiTNF-α therapy alone in patients with psoriatic arthritis [14]. Mader et al. from the Ha’emek Medical Center in Afula have shown the utility of musculoskeletal ultrasound in identifying entheseal changes in diffuse idiopathic skeletal hyperostosis (DISH) [15].

Shaare-Zedek Medical Center researchers, in collaboration with the Hadassah Medical Center investigators, have demonstrated lower overall incidence rates of vasculitis among the Jewish population despite rising incidence rates of granulomatosis with polyangiitis and microscopic polyangiitis [16]. In the same center, Mevorach et al. have demonstrated the utility, safety, and efficacy of eculizumab in pediatric patients with recurrent acute, predominantly motor demyelinating neuropathy with conduction block, and chronic hemolysis attributed to p.Cys89Tyr mutation in the CD59 gene [17].

### Other Middle East countries

Generally, there is quantitative and qualitative improvement in rheumatology research produced from these countries in the last five decades, as well as collaborative research work between countries within the region [18]. We have retrieved 76,146 papers from the National Library of Medicine’s (NLM) MeSH database in rheumatology between 1997 and 2017. Of those, 4513 (5.9%) were published from these countries. Turkey has contributed 1947 papers, 43% of the papers derived from Middle East countries. On the other hand, some countries, such as Yemen, did not produce any paper over the last two decades.

The differences between Middle Eastern countries in the published research can be attributed to the number of rheumatologists and the number of academic institutes within a country, political stability, the availability of funds, research facilities, an appropriate environment to conduct research, and the incentives for research.

In the last decade, governments in the Middle East have created research grants and awards to facilitate research. Pharmaceutical industries are vital partners in this transformation by spreading research skills among Middle East rheumatologists and providing funds for research. Furthermore, healthcare systems in Middle Eastern countries have an important role to play in promoting research culture, allocating time and incentive for those involved in research. Needless to say, it is important to mention that optimizing collaborative research projects between Middle Eastern countries will help in producing high-quality research from the region [18].

## Conclusion

The rheumatologists in the Middle East strive to improve the care of patients with rheumatic diseases despite the prevailing challenges in some countries. A few countries in the Middle East have contributed, and will continue to contribute, research and knowledge in the field of rheumatology. There is a great opportunity to improve research in some countries to reflect the high standard of care provided within the region.

## Abbreviations

ASIA: Autoimmune/inflammatory syndrome induced by adjuvant
CRP: C-reactive protein
DISH: Diffuse idiopathic skeletal hyperostosis
DMARD: Disease-modifying antirheumatic drug
FMF: Familial Mediterranean fever
IL: Interleukin
SLE: Systemic lupus erythematosus
TNF: Tumor necrosis factor
